# Photocatalytic Degradation of Carbofuran in Water Using Laser-Treated TiO_2_: Parameters Influence Study, Cyto- and Phytotoxicity Assessment

**DOI:** 10.3390/toxics12080566

**Published:** 2024-08-03

**Authors:** Miloš Tošić, Jasmina Savić, Ana Valenta Šobot, Sanja Živković, Aleksandra Dimitrijević, Nevena Ilić, Suzana Dimitrijević-Branković, Miloš Momčilović

**Affiliations:** 1 Department of Physical Chemistry, Vinča Institute of Nuclear Sciences, University of Belgrade, Mike Petrovića Alasa 12–14, 11000 Belgrade, Serbia; milos.tosic@vin.bg.ac.rs (M.T.); jasnas@vin.bg.ac.rs (J.S.); ana_v.s@vin.bg.ac.rs (A.V.Š.); sanjaz@vin.bg.ac.rs (S.Ž.); daleksandra@vin.bg.ac.rs (A.D.); 2 Innovation Centre, Faculty of Technology and Metallurgy, University of Belgrade, Karnegijeva 4, 11000 Belgrade, Serbia; nilic@tmf.bg.ac.rs; 3 Department of Biochemical Engineering and Biotechnology, Faculty of Technology and Metallurgy, University of Belgrade, Karnegijeva 4, 11000 Belgrade, Serbia; suzana@tmf.bg.ac.rs

**Keywords:** photocatalytic degradation, carbofuran, laser-treated TiO_2_, parameters study, cytotoxicity, phytotoxicity

## Abstract

This study investigates the impact of changing parameters on the photocatalytic degradation of carbofuran (CBF) using laser-treated TiO_2_ nanotube arrays on a Ti mesh under simulated sunlight irradiation and assessing toxicity during photocatalytic degradation. Various parameters, including the stirring effect, light intensity, initial CBF concentration, and variation in the active surface area of laser-treated TiO_2_ photocatalysts, were examined to determine their impact on degradation efficiency. The photodegradation kinetics were monitored using ultra-performance liquid chromatography with a PDA detector (UPLC-PDA) and UV-Vis spectrophotometry, while mineralization was assessed by a total organic carbon (TOC) analyzer. The photocatalytic degradation of CBF is enhanced by an increase in the active surface area of the TiO_2_ photocatalyst, light intensity, and the introduction of stirring, but it decreases with an increase in the initial concentration of CBF. The toxicity assessments revealed that the cytotoxicity of CBF initially increased during the degradation process but decreased after further treatment, indicating the formation and subsequent breakdown of toxic intermediates. The phytotoxicity test showed that longer degradation times resulted in higher toxicity to plant growth. This study provides new insights into the photocatalytic degradation of CBF with TiO_2_, the importance of parameter optimization for more efficient treatment, and the use of toxicity tests to confirm the success of the photocatalytic process.

## 1. Introduction

As a result of the escalating environmental contamination, advancements are being made in ecological purification technology. Conventional purification processes such as flotation, coagulation, chemical precipitation, oxidation, and adsorption are unable to completely eliminate all pollutants, often due to their low concentrations. Additionally, these processes have limitations, such as the requirement of hazardous chemicals, electricity, or other energy sources. For example, coagulating or flocculating uses a large quantity of chemicals and generates a huge volume of sludge, flotation consumes a large amount of electricity, and chemical precipitation requires chemical consumption (lime, oxidants, H_2_S, etc.) [1,2]. Advanced oxidation processes (AOPs) offer an efficient solution due to their relatively low cost, environmentally friendly, sustainable treatment technology, and ability to overcome the shortcomings of conventional technologies. TiO_2_ is a powerful part of AOPs, which can effectively oxidize a wide range of organic contaminants through reactive oxygen species (ROS) [3,4].

Many studies demonstrate that TiO_2_ powder has the ability to catalyze the photodegradation of various pollutants [5,6]. However, a challenge arises when incorporating slurry in water purification, as it necessitates additional steps to separate TiO_2_ from purified water after degradation. If the concentration of TiO_2_ particles exceeds a certain threshold, it can negatively affect the efficiency of degradation [7]. Also, TiO_2_ photocatalyst particles can aggregate, influencing the optical properties of materials such as absorption and scattering of irradiation, thereby affecting the efficiency of photocatalytic processes [8,9]. To overcome these drawbacks, a viable solution can be the use of TiO_2_ nanotube arrays formed on the Ti substrate. Electrochemical anodization is the most straightforward path to obtain TiO_2_ nanotube arrays with controllable dimensions through self-organization [10]. Layers form on the metal surface, enabling semiconductor formation on the Ti substrate without additional immobilization [11,12]. Using Ti mesh to create TiO_2_ nanotube arrays for pollutant degradation is becoming more prevalent. Liquid pollutants may easily diffuse through porous materials, which increases degradation efficiency. Ti mesh results in a much better light utilization rate than the Ti plate and foil and can be used as a novel electrode material to remove pollutants from water [13,14,15,16]. Our previous study was aimed at improving the photocatalytic properties of TiO_2_ on Ti mesh by the synergistic effect of Nd:YAG picosecond pulsed laser irradiation and electrochemical anodization to form TiO_2_ nanotube arrays with Ti^3+^/oxygen vacancies and anatase/rutile phase [17]. Although there have been many studies on the degradation of CBF using TiO_2_ photocatalyst as a part of AOPs [18,19,20], to the best of our knowledge, no research has been conducted on CBF degradation using TiO_2_ nanotube arrays on a Ti substrate except our previous initial study [17], and this one in which we investigated the influence of various parameters on CBF degradation using laser-treated TiO_2_ nanotube arrays on a Ti substrate.

Carbofuran is an N-methylcarbamate pesticide that is widely used in agriculture as an insecticide and nematicide and is considered as highly hazardous pesticide. It has high mobility in soils and is highly soluble in water (700 mg/L) [21,22]. Its usage is prevalent in Asia, Australia, and South America, while Canada, Kenya, Brazil, the United States, and the European Union have implemented bans or restrictions on the use of CBF [23]. In Africa, CBF is most frequently misused [24]. In Serbia, CBF was banned in 2014. However, since it still occurs sporadically due to illegal and unconscionable use in already prohibited countries, it can be concluded that the complete cessation of its use and harmful occurrences will require an additional period [22,25,26,27,28]. Therefore, it is still important to pay attention to its more efficient removal from the environment. CBF, like other pesticides, can be derived to aquatic environments from several sources, including direct application, runoff from agricultural land, wastewater discharges, and atmospheric deposition during the application process, which can subsequently contaminate streams through dry or wet deposition [29]. CBF is a highly toxic pesticide when inhaled or ingested and moderately toxic when absorbed dermally, affecting nervous system functions [30]. The mechanism of action of CBF is mainly through the reversible inhibition of the acetylcholinesterase enzyme (AChE), followed by endocrine disruption [31]. According to the World Health Organization (WHO), CBF is classified as extremely hazardous to humans (WHO 1b) due to its low acute oral rat LD_50_ values, which can be as low as 5 mg/kg [23].

Commonly, the parent pesticide compounds are harmful, and degradation reduces their toxicity. In certain circumstances, degradation may yield products more toxic than the parent compounds. This phenomenon can be elucidated by the subsequent explanations. First, products may contain harmful active ingredients and use the same mode of action as the parent compounds. Second, changes in molecular weight and hydrophobicity may increase the bioconcentration of products compared to their parent compounds, the distribution of more hazardous substances, or their interaction with target receptors. And thirdly, the emergence of products with different modes of toxic action. Therefore, it is crucial to examine the toxicity of pesticides during decomposition [32].

The objective of this study was to evaluate the applicability of a laser-treated TiO_2_ photocatalyst for pollutant photodegradation. As a model system for this testing, CBF degradation in an aqueous solution was examined, and the experimental parameters for CBF photocatalytic degradation were studied. The stirring effect, variation in the photocatalyst active surface area, simulated sunlight irradiation intensity, and initial CBF concentration are some of the parameters. The cytotoxicity and phytotoxicity of a photodegradation solution consisting of parent compounds and photodegradation products have been evaluated with regard to process time.

## 2. Materials and Methods

### 2.1. Reagents and Chemicals

Carbofuran (C_12_H_15_NO_3_, 99,65%) was purchased from Dr. Ehrenstorfer, LGC Standards Ltd. (Augsburg, Germany), titanium mesh (Ti) from Goodfellow (Wrexham, UK), ethylene glycol (C_2_H_6_O_2_, 99.5%), isopropyl alcohol (C_3_H_8_O, 99.5%), from Centrohem (Stara Pazova, Serbia), nitrogen gas 5.0 (N_2_) from Messer Technogas (Bad Soden, Germany), formic acid (CH_2_O_2_, 99%) from Carlo Erba (Emmendingen, Germany), acetonitrile—HPLC Gradient Grade (C₂H₃N) from J.T. Baker (Randor, PA, USA), and ammonium fluoride—pro analysis (NH_4_F, 98%) was obtained from Merck (Rahway, NJ, USA). The 2,3-Bis(2-methoxy-4-nitro-5-sulfophenyl)-2H-tetrazolium-5-carboxanilide inner salt (C_22_H_16_N_7_NaO_13_S_2_) (XTT), phenazine methosulfate (C_14_H_14_N_2_O_4_S) (PMS), and phosphate buffered saline (PBS) used in viability assays were produced by Serva (Heidelberg, Germany).

### 2.2. Synthesis of Photocatalysts

The laser-treated TiO_2_ photocatalyst was synthesized as previously described in our study [17]. The Ti mesh underwent successive 10 min cleanings in acetone, ethanol, and distilled water by sonication. After cleaning, electrochemical and chemical polishing of the photocatalyst were carried out. TiO_2_ synthesis was conducted by irradiating a titanium mesh with a pulsed laser in the air at atmospheric pressure and room temperature. Irradiation was performed with a picosecond Nd:YAG laser (Ekspla, Vilnius, Lithuania) (Ekspla SL 212/SH/FH, 150 ps) operating at a wavelength of 1064 nm and a repetition rate of 10 Hz at a fluence of 30 J/cm^2^. During the irradiation, the laser was focused on the Ti target using a 15.2 cm lens. The Ti mesh was precisely ablated using a motorized *XY* translation stage. The Ti mesh surface was scanned until an irradiated surface of 1 cm^2^ was achieved. The Ti mesh was irradiated from both sides under identical conditions. The sample was then subjected to electrochemical anodization to form nanotube arrays and calcination. The equivalent procedure was implemented to yield samples with a total active surface of 2, 3, or 4 cm^2^. Experiments that were performed in the presence of sample surfaces of 1, 2, 3, and 4 cm^2^ are marked as 1-L-TiO_2_, 2-L-TiO_2_, 3-L-TiO_2_, and 4-L-TiO_2_, respectively.

### 2.3. Photocatalytic Process and Modifications of Parameters

#### 2.3.1. Photodegradation Reactor

For the photodegradation studies, an open reactor with a double-walled cooling water jacket was used to maintain a constant temperature. An irradiation lamp (Osram Ultra-vitalux 300 W, Osram, Munich, Germany) simulating sunlight was held at a distance of about 10 cm. Next, 20 mL of CBF solution was irradiated after the first 30 min in the dark to achieve adsorption-desorption equilibrium. The reaction solutions taken from the photoreactor after 30 min in the dark and 30, 60, 90, 120, and 150 min of irradiation were used for further tests (chromatographic separation, spectrophotometric analysis, TOC analyzer analysis, and toxicity testing). The light intensity of the irradiation lamp was modified using a variable transformer (0–260 V), whose voltages of 185 V, 215 V, and 235 V correspond to light intensities of 500, 1100, and 1300 W/m^2^, respectively. Modified voltages were monitored during photocatalysis with a Voltcraft 2010 analog multimeter, while initial and final light intensities were controlled with a Voltcraft PL-110SM luxmeter. Both instruments were purchased from Voltcraft (Wollerau, Germany).

#### 2.3.2. Modifications of Experimental Parameters

The applicability of the synthesized TiO_2_ was tested in CBF degradation, which was investigated as a function of photocatalyst active surface area, initial CBF concentration, light intensity, and with or without stirring in the photocatalytic process. Investigation of experimental parameters for photocatalytic degradation included modifying one parameter while keeping the others unchanged. The ranges of experimental parameters for investigation of CBF photocatalytic degradation kinetics for catalyst area were from 1 to 4 cm^2^ (1-L-TiO_2_, 2-L-TiO_2_, 3-L-TiO_2_, and 4-L-TiO_2_ samples), for initial CBF concentrations of 2.5, 5, 10, and 15 mg/L, and for light intensities of 500, 1100, and 1300 W/m^2^. CBF solutions were prepared using deionized water.

### 2.4. Analytical Methods and Performance Indices

The progress of CFB photodegradation was followed by UPLC-PDA (Waters Acquity, Milford, MA, USA) and Lambda 35 UV-Vis spectrophotometer (Perkin Elmer, Shelton, CT, USA). The CBF degradation [%] was calculated by Equation (1) [17]:

(1) $$Degradation\;\left(\%\right)=\frac{C_{0}-C}{C_{0}}\times 100$$

where *C*_0_ and *C* represent the initial and CBF concentration at time *t*, respectively.

The kinetics of CBF degradation/mineralization was evaluated with the pseudo-first order reaction model from Equation (2) [33]:

(2) $$-\ln\left(\frac{C}{C_{0}}\right)=k_{1}t$$

where *k*_1_ is the pseudo-first order rate constant.

To facilitate the analysis and conclusion of CBF and product toxicity, samples taken at different time intervals during the photocatalytic process of 15 mg/L CBF in the presence of 4-TiO_2_ and 1300 W/m^2^ were quantified by the UPLC-PDA system. The UPLC-PDA conditions for the analysis are detailed in the Supplementary Information (Table S1).

TOC-V analyzer (Shimadzu, Kyoto, Japan) assessed TOC concentration. The remaining mineralization [%] of CBF reaction solutions under optimal experimental conditions was determined based on TOC measurements from Equation (3):

(3) $$Remaining\;mineralization\;\left(\%\right)=\frac{{TOC}_{0}-TOC}{{TOC}_{0}}\times 100\;$$

where *TOC*_0_ and *TOC* represent the initial and concentration after time *t*, respectively. The pH of the solutions was determined using an Orion Star A111 pH meter (Thermo Scientific, Waltham, MA, USA).

### 2.5. Practical Application, Stability, Reusability, and Effect of Various Scavengers on Photocatalyst Performance

The applicability of the synthesized TiO_2_ photocatalytic degradation of CBF in tap and river water was studied for practical use. A sample of river water was collected from the Danube River (Vinča, Serbia). Tap and river water were spiked with 15 mg/L CBF before photocatalytic degradation. Photocatalyst stability and reusability are crucial for its applicability. Five photocatalytic degradation experiments were performed at pH = 7.0, [CBF]_0_ = 15 mg/L, and a light intensity of 1300 W/m^2^ to test the 4-L-TiO_2_ sample photocatalytic stability and reusability. To investigate the primary mechanism responsible for 15 mg/L CBF degradation during the photocatalytic process with 4-L-TiO_2_, and 1300 W/m^2^ light intensity, the removal of CBF was observed in the presence of isopropyl alcohol (IPA) 5 mM, formic acid (FA) 5mM, and N_2_ as ^•^OH, h^+^, and ^•^O_2_¯ scavengers, respectively [34,35,36].

### 2.6. Toxicity Experiments

#### 2.6.1. Cytotoxicity Experiment—Cell Culture and Treatment

The human fibroblasts (MRC-5 cell line) were seeded in a 24-well plate at 0.05 × 10^6^ cells per well and cultured in 900 µL of Dulbecco’s Modified Eagle’s Medium (DMEM, Gibco, Thermo Fisher Scientific, Norristown, PA, USA) supplemented with 10% heat-inactivated fetal bovine serum (FBS, Capricorn Scientific, Ebsdorfergrund, Germany) at 37 °C and 5% CO_2_. After 24 h of cultivation, at the subconfluent level, 300 µL of cultivation media was replaced with 15 mg/mL CBF solution (initial solution and reaction solutions after 30, 90, and 150 min of irradiation in the photoreactor). At the end of the 24 h treatment period, XTT assay was performed [37].

#### 2.6.2. Phytotoxicity Experiment

To evaluate the toxicity of 30% CBF and its degradation products at different time intervals (control, 0, 30, 90, and 150 min), a phytotoxicity test was performed using wheat seeds (*T. aestivum*) [38].

The seeds were chemically sterilized in a 70% ethanol and 6% NaClO solution, then washed with sterile distilled water. Fifteen seeds were incubated in each sample. After that, the seeds were placed in sterile Petri dishes with filter paper, treated with 1 mL of sterile distilled water to initiate germination, and incubated for 4 days at 25 °C in the dark. A visible root was used as the operational definition of seed germination, and the length of the roots was measured. Based on these data, the relative root length value (RLV), relative germination value (RGV), and germination index (GI) were calculated.

*RLV* was calculated according to Equation (4):

(4) $$RLV\;\left(\%\right)=\left(\frac{{ALV}_{s}}{{ALV}_{c}}\right)\times 100$$

where *RLV* is relative root length value [%], *ALV_S_* [cm] is the average root length value in the sample, *ALV_C_* [cm] is the average root length value in the control. *RGV* was calculated according to Equation (5):

(5) $$RGV\;\left(\%\right)=\left(\frac{{NGS}_{s}}{{NGS}_{c}}\right)\times 100$$

where *RGV* is the relative germination value [%], *NGS_S_* is the number of germinated seeds in the sample, and *NGS_C_* is the number of germinated seeds in the control. *GI* was calculated according to Equation (6):

(6) $$GI\;\left(\%\right)=\left(\frac{RLV}{RGV}\right)\times 100$$

where *GI* is germination index value [%], *RLV* is relative root length value [%], and *RGV* is relative germination value [%].

## 3. Results

### 3.1. Modifications the Experimental Parameters for Photocatalytic Degradation of CBF

#### 3.1.1. Stirring Effect

**Figure 1:**
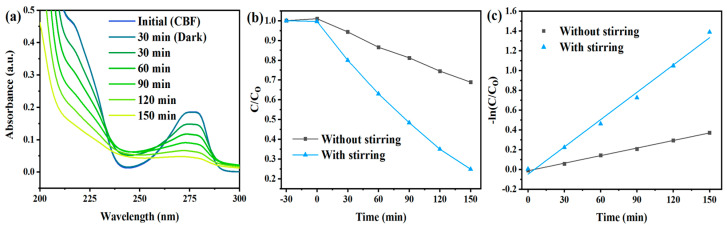
(**a**) Photocatalytic degradation of CBF with stirring followed by UV-Vis. (**b**) Comparison of the photodegradation rate and (**c**) kinetic constant with and without stirring. Experimental condition: 4-L-TiO_2_; 1300 W/m^2^; [CBF]_0_ = 15 mg/L; pH = 7.0.

The experiment aimed to investigate the effect of stirring on the photocatalytic degradation of CBF. CBF degradation was followed by UV-Vis spectrophotometry (Figure 1a).

Figure 1b indicates that stirring enhances the photocatalytic degradation of CBF, and that the % of photocatalytic degradation with and without stirring is equal to 75.1% and 31.1%, respectively. The kinetic constants of degradation without and with stirring (Figure 1c) are 0.0025 min^−1^ (R^2^ = 0.9986) and 0.0092 min^−1^ (R^2^ = 0.9919), respectively. Stirring may actually increase the interaction of ROS with pollutant molecules. Therefore, further experiments incorporated stirring.

#### 3.1.2. Effect of Light Intensity

**Figure 2:**
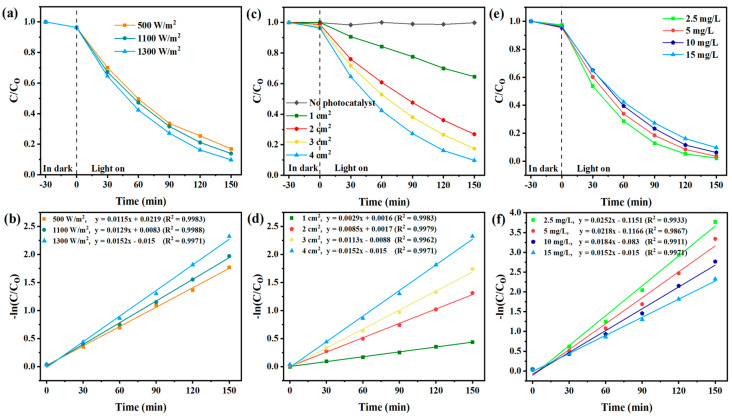
Photocatalytic degradation of CBF and kinetic rates obtained with UPLC-PDA with different: (**a**,**b**) light intensities, (**c**,**d**) TiO_2_ photocatalyst active surface area, (**e**,**f**) initial CBF concentration.

Figure 2a demonstrates that increasing light intensity, i.e., the reaching of more photons in a given period, promotes degradation efficiency and accelerates photocatalytic activity [39]. Increasing light intensity results in the availability of more energy required for the generation of e^−^/h^+^ and ROS production. This, in turn, accelerates the degradation process (Figure 2a,b) [40]. Therefore, a light intensity of 1300 W/m^2^ was selected as optimal.

#### 3.1.3. Effect of TiO_2_ Photocatalyst Active Surface Area

Generally, the greater the surface area of the photocatalyst, the more active sites are accessible for photocatalyst interactions, generating more ROS and leading to a higher degradation rate. In the absence of photocatalysts or simulated sunlight irradiation, control experiments showed that the concentration of CBF remained constant, suggesting that CBF was very stable and that degradation on its own was negligible (Figure 2c,d).

#### 3.1.4. Effect of Initial CBF Concentration

**Figure 3:**
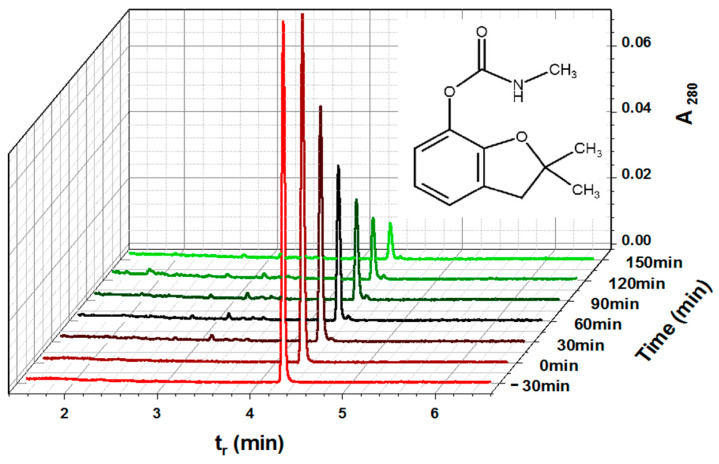
Photocatalytic degradation of CBF followed by UPLC-PDA. Experimental condition: 4-L-TiO_2_; 1300 W/m^2^; [CBF]_0_ = 15 mg/L; pH = 7.0.

As CBF concentration increased, photocatalytic degradation efficiency decreased (Figure 2e). CBF degradation was the highest at 2.5 mg/L and the lowest at 15 mg/L (Figure 2f). It is well known that a higher pollutant initial concentration results in its excessive adsorption on the photocatalyst surface, preventing photocatalyst activation [41]. As a result, insufficient generation of reactive oxygen species during irradiation reduces the effectiveness of pollutant photodegradation [42,43]. It can be concluded that the presence of a higher CBF concentration in the solution can decrease the photocatalytic reactions and rate constants. Figure 3 shows UPLC chromatograms of initial CBF solutions (−30 min and 0 min) and CBF reaction solutions taken from the photoreactor after 30–150 min of irradiation.

Table 1 contains CBF degradations determined based on chromatographic and spectrophotometric data for different experimental parameters.

**Figure 4:**
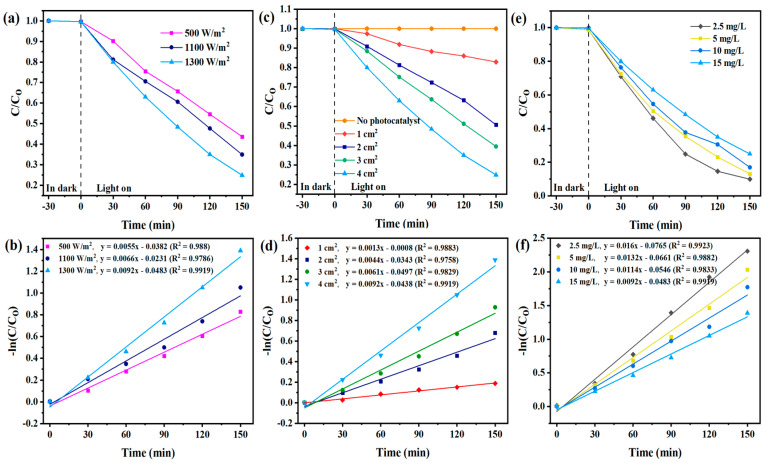
Photocatalytic degradation of CBF and kinetic rates obtained with UV-Vis with different: (**a**,**b**) light intensities, (**c**,**d**) TiO_2_ photocatalyst active surface area, (**e**,**f**) initial CBF concentration.

**Figure 5:**
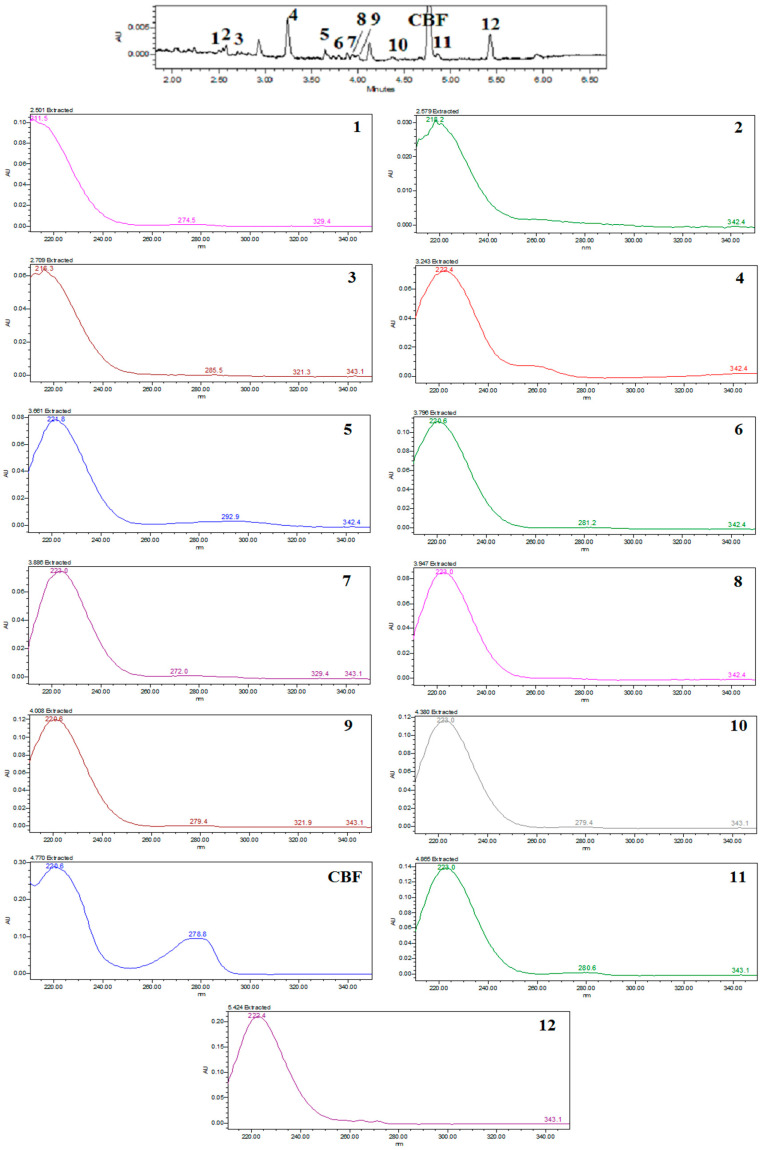
2D chromatogram at 280 nm and PDA spectra of components from reaction solution taken from photoreactor after 90 min of irradiation (reaction solution was concentrated before chromatographic separation).

UV-Vis spectrophotometry yields comparable results and leads to similar conclusions when examining the impact of variations in operating parameters (Figure 4a–f), with more noticeable distinctions between different parameter values presented in Table 1. This can be attributed to the presence of CBF degradation products, which contribute to the overall recorded absorption spectrum of the degradation solution together with the parent compound (Figure 5).

**Figure 6:**
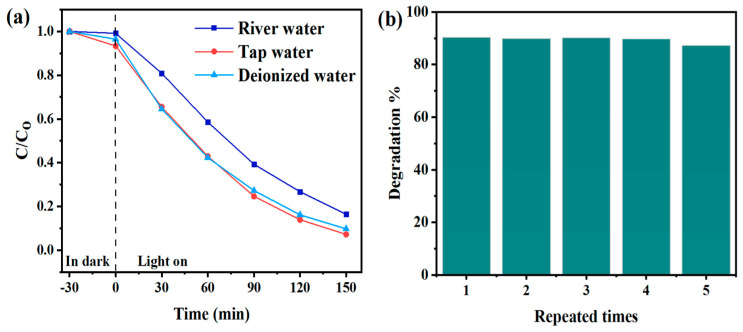
(**a**) Photocatalytic degradation of CBF carried out in deionized, tap, and river water, (**b**) reusability experiments.

Figure 6a shows the degradation rates of deionized (pH 7.0), tap (pH 7.5), and river water (pH 7.9). Degradation of 15 mg/L CBF in tap and deionized water samples at the same experimental parameters (4-L-TiO_2_ and light intensity of 1300 W/m^2^) are similar, i.e., 92.7% and 90.3%, respectively. The degradation extent of 15 mg/L CBF in river water is equal to 83.5%. Since it is lower than for tap and deionized water, that indicates hindered 4-L-TiO_2_ activity, which may be due to more complex natural organic matter or inorganic ions, as previously reported [44,45]. Photocatalytic activity decreases from 90.3% to 87.2% after the fifth run. These tests were conducted after 30 min in the dark for each run. After each test, 4-LTiO_2_ was cleaned in pure water for 30 min by immersing, and then dried at room temperature. For CBF photodegradation, 4-L-TiO_2_ is stable and reusable due to its high degradation efficiency (Figure 6b).

### 3.2. Effect of Scavengers on Photocatalyst Performance

**Figure 7:**
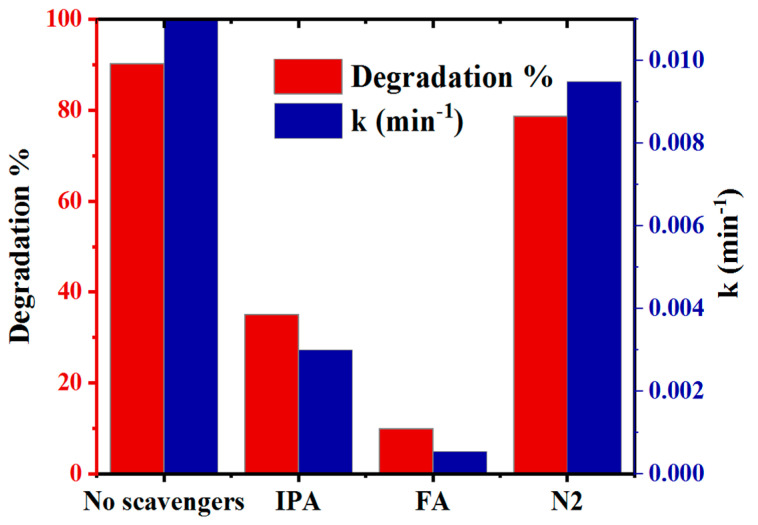
Effects of various scavengers on photocatalytic degradation of CBF and their rate constant values.

The addition of each of the three scavenging agents (IPA, FA, and N_2_) reduced the rates of CBF degradation efficiencies from 90.3% to 35.1% (0.003 min^−1^), 9.9% (5.36×10^−4^ min^−1^), and 78.7% (0.0095 min^−1^), respectively. The addition of IPA and N_2_ to the initial CBF solution inhibited its photodegradation, which indicated that ^•^OH and ^•^O_2_¯ contribute modestly to the removal of CBF. Since the degradation efficiency decreased by 84.1% in the case of FA addition, which indicated that h^+^ was the primary oxidative species in the photocatalytic degradation of CBF (Figure 7). Under simulated sunlight irradiation, the conduction band generates an electron e^−^(CB) by exciting an electron from the valence band, which forms a positively charged hole h^+^(VB) (Equation (7)), oxidizing H_2_O on the surface to produce a hydroxyl radical (^•^OH) (Equation (8)), while molecular oxygen adsorbs on the surface, trapping electrons in the conduction band and producing a superoxide anion radical by the reduction reaction (Equation (9)) [46]. Ti^3+^/oxygen vacancies enhance electron transport and minimize recombination of e^−^/h^+^ pairs by creating a sublevel potential below the CB of 4-L-TiO_2_ [17]. Hence, the positively charged holes, hydroxyl, and superoxide radicals participate in the photocatalytic degradation (Equation (10)).

4-L-TiO_2_ + hν → h^+^(VB) + e^−^(CB) (7)

h^+^(VB) + H_2_O → ^•^OH + H^+^ (8)

e^−^(CB) + O_2_ → ^•^O_2_^−^ (9)

CBF + h^+^/^•^OH/^•^O_2_^−^ → Degradation products + H_2_O + CO_2_ + NH_4_^+^ (10)

### 3.3. Mineralization of CBF

**Figure 8:**
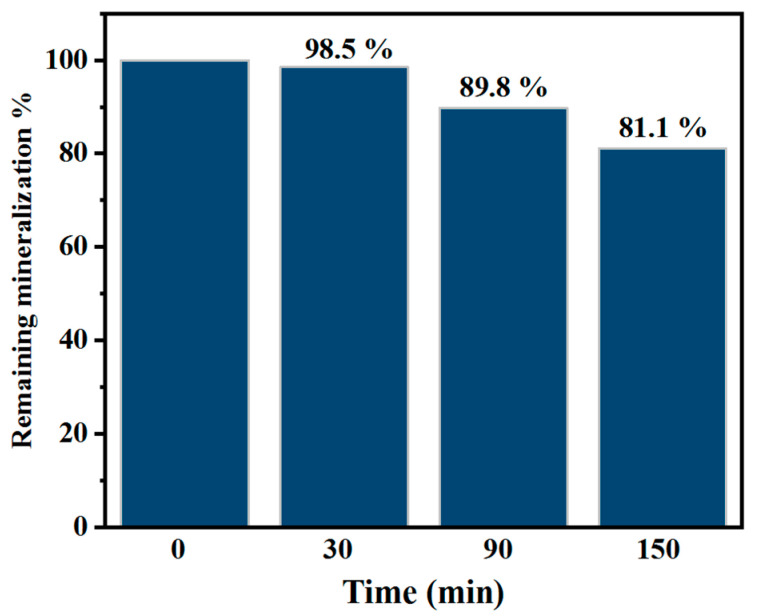
Mineralization of CBF. Experimental condition: 4-L-TiO_2_; 1300 W/m^2^; [CBF]_0_ = 15 mg/L; pH = 7.0.

Figure 8 shows photocatalytic CBF mineralization results. As previously stated, the procedure degraded 90.3% of CBF in 150 min and mineralized 18.9%. The production of simpler organic intermediates during CBF degradation may explain its slower mineralization than degradation. The disparity between degradation and mineralization rates shows that CBF degradation intermediates remain in the solution and could be mineralized by extending reaction time.

According to this and prior studies, well-aligned, highly ordered, and modified TiO_2_ nanostructures can improve light absorption and charge separation, improving photocatalytic activity. Table 2 reviews and compares TiO_2_ nanostructures on Ti substrates for photocatalytic pollutant degradation.

**Table 2:** Photocatalytic degradation of various pollutants with TiO_2_ nanostructures formed on Ti substrate.

Substrate/Area		Pollutant/Concentration	Degradation	Light Source	Ref.
TiO_2_ (Ti foil)	Barrier Nanotubular Mixed/1 cm^2^	Polystyrene nanoparticles/ 0.9% *w/v*	16.2% 19.7% 23.5%	50 h/UV light, 0.021 mW/cm^2^	[47]
TiO_2_ (Ti foil)	Nanotube arrays/1 cm^2^	Rhodamine B/10 μM Bisphenol A/50 μM	88% 36%	300 min/UV-A light, 2 mW/cm^2^	[48]
TiO_2_ (Ti mesh)	Nanotube arrays/6.25 cm^2^	Toluene/10 ppm	90%	30 min/UV-A light 15 W	[49]
TiO_2_ (Ti foil)	Nanotube arrays/3 cm^2^	Methyl orange/16.4 mg/L	97.29%	240 min/Xe lamp 10 mW/cm^2^	[50]
TiO_2_ (Ti mesh)	Nanotube arrays/6.75 cm^2^	Chloramphenicol/10 mg/L	91%	120 min/Xe lamp 300 W/Visible light	[51]
TiO_2_ (Ti mesh)	Nanotube arrays/4 cm^2^	Carbofuran/15 mg/L	90.3%	150 min/Sunlight simulation 300 W, 1300 W/m^2^	This study

### 3.4. Effect of Photocatalytic Treatment of CBF on Cytotoxicity and Phytotoxicity

Cytotoxicity and phytotoxicity tests used a photocatalytically treated CBF solution of 15 mg/L under the following parameters: 4-L-TiO_2_, light intensity of 1300 W/m^2^, and stirring. Toxicity tests were performed at 0, 30, 90, and 150 min, with CBF concentrations of 15, 9.4, 4.0, and 1.5 mg/L in reaction solutions, respectively. For easier comparison and analysis, the samples taken at different time intervals were quantified by UPLC-PDA, and a calibration curve for CBF was created.

**Figure 9:**
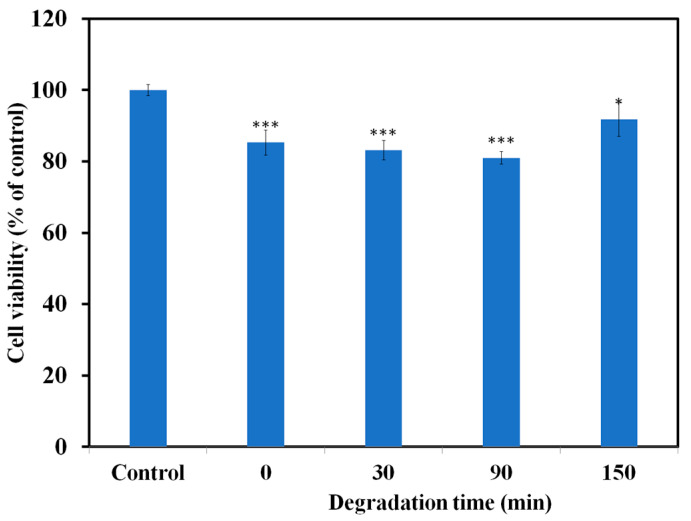
Cell viability following 24 h treatment with CBF and its degradation products after 0, 30, 90, and 150 min. Results are presented as relative values to the control and significant difference marked as * *p* < 0.05; *** *p* < 0.001.

Compared to the control group, Figure 9 demonstrates the cytotoxicity of fresh CBF solution (15 mg/L) and treated solution at various time interval degraded solutions taken from the photoreactor after different irradiation times. Figure 9 shows that CBF is cytotoxic, as cell viability decreased from 100% (control) to 85.3%, 83.1%, and 80.9% when cells were exposed to fresh solutions sampled at 0, 30, and 90 min, respectively. This can be attributed to the formation of CBF intermediates such as 3-Hydroxycarbofuran, 3-Ketocarbofuran, and others with toxic active moieties with different or the same mode of action as CBF [20,52,53,54,55]. Despite efficient CBF degradation (90.3%) at the end of photocatalytic treatment, cytotoxicity remains (cell viability 91.7%), albeit to a lesser extent, indicating CBF molecules are broken down into less cytotoxic end products.

The phytotoxicity of the tested samples was evaluated based on the germination index (GI). According to the criteria, samples with a GI between 25% and 65% indicate phytotoxicity, those with a GI higher than 65% indicate non-phytotoxicity, and those with a GI higher than 101% indicate a phytostimulating effect [38].

The results of both cytotoxicity (Figure 9) and phytotoxicity tests (Table 3) provide a comprehensive understanding of the effects of photocatalytically treated CBF and its degradation products. The phytotoxicity tests using wheat seeds (*T. aestivum*) revealed that the toxicity of the samples increased with longer degradation times. At 0 min, GI of 96.56% indicated that the sample was non-phytotoxic, with a relative root length value (RLV) of 181.04% suggesting significant root growth compared to the control. However, as the degradation time increased, the phytotoxic effects became more pronounced. At 30 min, the GI decreased to 46.25%, indicating phytotoxicity, with an RLV of 77.08%, showing reduced root growth. At 90 min, the GI further dropped to 13.41%, indicating high phytotoxicity, with an RLV of 50.27%, showing significant inhibition of root growth. At 150 min, the GI remained low at 13.91%, also indicating high phytotoxicity, with an RLV of 52.17%, showing similar inhibition to the 90 min sample. These results suggest that the degradation products formed at longer treatment times are more toxic to plant growth.

The combined results from the phytotoxicity and cytotoxicity tests indicate a complex relationship between the degradation of CBF and its resulting toxicity. While photocatalytic treatment effectively reduces the overall concentration of CBF and its acute cytotoxicity, the formation of intermediate degradation products significantly increases phytotoxicity over time, which possess toxic active moieties and maintain the same mode of action as the original CBF, contributing to the observed phytotoxic effects.

The increasing phytotoxicity with longer degradation times highlights the importance of not only reducing the concentration of the original contaminant but also ensuring that intermediate products are sufficiently broken down into non-toxic end products. Although the photocatalytic process significantly decreases acute cytotoxicity, the remaining toxicity from intermediate compounds suggests the need for extended treatment durations or additional remediation steps to fully neutralize harmful effects. For instance, Lopez-Alvarez et al. found that degradation products of CBF retained about 30% of toxicity (tested on *Vibrio fischeri* bacteria) after 150 min of photocatalytic degradation, which declined to greater than an 80% reduction in toxicity by the end of treatment after 320 min [53].

The discrepancy between the degradation and mineralization rates in our study indicates that while carbofuran is broken down into simpler compounds, these intermediates are not fully mineralized into non-toxic end products within the studied timeframe. These findings underscore the need for comprehensive toxicological evaluations when applying AOPs for pollutant degradation. It is not sufficient to assess the effectiveness of AOPs based solely on the removal of the parent compound. Understanding the toxicity profiles of the intermediate products is crucial to ensure that the treatment process does not inadvertently introduce new environmental hazards.

## 4. Conclusions

In this study, the influence of various parameters, such as the total active surface area of the laser-treated TiO_2_ photocatalyst, stirring effect, light intensity, and initial CBF concentration on CBF photodegradation was investigated using analytical techniques (UPLC-PDA and UV-Vis) during the photocatalytic degradation process of CBF. The photodegradation of CBF is enhanced by an increase in the active surface area of the TiO_2_ photocatalyst, light intensity, and the introduction of stirring, but it decreases with an increase in the initial concentration of CBF. Key results demonstrated a maximum degradation of 90.3% for 15 mg/L CBF at a light intensity of 1300 W/m^2^ and a TiO_2_ photocatalyst active surface area of 4 cm^2^ with stirring after 150 min of simulated sunlight irradiation (optimal parameters). The trapping experiments showed that the photocatalytic degradation of CBF is mainly caused by h^+^, and modestly ^•^OH and ^•^O_2_¯. Toxicity studies were carried out to verify the efficacy of degradation under optimal parameters. The results of the tests showed complex toxicity during CBF degradation. It was shown that cytotoxicity initially increases during the photocalytic degradation process and then decreases, while phytotoxicity increases with time. The efficacy of AOPs in eliminating toxic compounds should be evaluated in tandem with the toxicological assessment analysis. Determining the efficacy of AOPs based only on the target compound degradation percentage is insufficient and potentially deceptive. The energy used in these processes can be significantly reduced by using natural sunlight, which is of the greatest interest from an economic and environmental point of view. This research offers an alternative method based on innovative laser and nanotechnologies for creating an effective photocatalyst that could be used for wastewater treatment.

## Figures and Tables

**Table 1:** CBF degradations depending on experimental parameters.

Unchanged Parameters	Modified Parameter	CBF Degradation (%)	
[CBF]_0_ = 15 mg/L Active surface area 4 cm^2^	Light intensity (W/m^2^)	UPLC-PDA	UV-Vis
	1300	90.3	75.1
	1100	86.1	65.1
	500	83.1	56.3
[CBF]_0_ = 15 mg/L Light intensity 1300 W/m^2^	Active surface area (cm^2^)	UPLC-PDA	UV-Vis
	4	90.3	75.1
	3	82.5	60.6
	2	73.2	49.4
	1	35.5	17.2
Active surface area 4 cm^2^ Light intensity 1300 W/m^2^	[CBF]_0_ (mg/L)	UPLC-PDA	UV-Vis
	15.0	90.3	75.1
	10.0	93.7	83.1
	5.0	96.5	86.9
	2.5	97.8	90.1

**Table 3:** Phytotoxicity parameters for CBF degradation during CBF photocatalytic treatment.

	Total Average Length of the Roots [cm] ± SD	RGV (%)	RLV (%)	GI (%)
Control *	1.32 ± 0.43	100.00	100.00	100.00
0 min	2.39 ± 0.32	53.33	181.04	96.56
30 min	1.02 ± 0.33	60.00	77.08	46.25
90 min	0.66 ± 0.23	26.67	50.27	13.41
150 min	0.69 ± 0.23	26.67	52.17	13.91

* Distilled water.

## Data Availability

Data are available upon reasonable request by the corresponding author.

## Supplementary Materials

The following supporting information can be downloaded at: https://www.mdpi.com/article/10.3390/toxics12080566/s1, Table S1: UPLC-PDA conditions.
