# Supplementary material for: Photocatalytic Degradation of Carbofuran in Water Using Laser-Treated TiO2: Parameters Influence Study, Cyto- and Phytotoxicity Assessment
Source: Toxics. 2024 Aug 3;12(8):566. doi: 10.3390/toxics12080566 (PMC11360000; doi:10.3390/toxics12080566)
Supplement: Supplementary file 1 [file toxics-12-00566-s001.zip › toxics-3131852-supplementary.pdf]

toxics

MDPI

Article

Photocatalytic Degradation of Carbofuran in Water Using Laser-Treated TiO_2: Parameters Influence Study, Cyto- and Phytotoxicity Assessment

Miloš Tošić [1], Jasmina Savić [1], Ana Valenta Šobot [1], Sanja Živković [1], Aleksandra Dimitrijević [1], Nevena Ilić [2], Suzana Dimitrijević-Branković [3] and Miloš Momčilović [1,*]

[1] Department of Physical Chemistry, Vinča Institute of Nuclear Sciences, University of Belgrade, Mike Petrovića Alasa 12–14, 11000 Belgrade, Serbia; milos.tosic@vin.bg.ac.rs (M.T.); jasnas@vin.bg.ac.rs (J.S.); ana_v.s@vin.bg.ac.rs (A.V.Š.); sanjaz@vin.bg.ac.rs (S.Ž.); daleksandra@vin.bg.ac.rs (A.D.)

[2] Innovation Centre of Faculty of Technology and Metallurgy, Karnegijeva 4, 1100 Belgrade, Serbia; nilic@tmf.bg.ac.rs

[3] Department of Biochemical Engineering and Biotechnology, Faculty of Technology and Metallurgy, University of Belgrade, Karnegijeva 4, 11000 Belgrade, Serbia; suzana@tmf.bg.ac.rs

* Correspondence: milos@vin.bg.ac.rs

Waters ACQUITY UPLC system with PDA detector and BEH C18 column (100 mm × 2.1 mm, 1.7 μm) as a stationary phase was used for chromatographic separation of reaction mixtures taken from photoreactor after certain irradiation times (0–150 min). All analyses were performed using the gradient condition (Table S1) with a mobile phase consisting of solvent A (0.1 wt% formic acid in water) and solvent B (0.1 wt% formic acid in acetonitrile) at a constant flow rate of 0.25 mL/min. The 3D data were recorded in the wavelength range from 210 to 350 nm, while 2D chromatograms were recorded at 275 and 280 nm. The run time was 7 min, and the injection volume was 5 μL. CBF retention time was 4.3 min.

Table S1. Gradient condition for UPLC separation of CBF reaction solutions obtained during the photocatalytic degradation process.

Time (min)	% B
0	40
4.5	77
5.5	97
5.6	40
7.0	40
